# Targeted deep sequencing analyses of long QT syndrome in a Japanese population

**DOI:** 10.1371/journal.pone.0277242

**Published:** 2022-12-08

**Authors:** Yuki Nagata, Ryo Watanabe, Christian Eichhorn, Seiko Ohno, Takeshi Aiba, Taisuke Ishikawa, Yukiko Nakano, Yoshiyasu Aizawa, Kenshi Hayashi, Nobuyuki Murakoshi, Tadashi Nakajima, Nobue Yagihara, Hiroyuki Mishima, Takeaki Sudo, Chihiro Higuchi, Atsushi Takahashi, Akihiro Sekine, Takeru Makiyama, Yoshihiro Tanaka, Atsuyuki Watanabe, Motomi Tachibana, Hiroshi Morita, Koh-ichiro Yoshiura, Tatsuhiko Tsunoda, Hiroshi Watanabe, Masahiko Kurabayashi, Akihiko Nogami, Yasuki Kihara, Minoru Horie, Wataru Shimizu, Naomasa Makita, Toshihiro Tanaka

**Affiliations:** 1 Bioresourse Research Center, Tokyo Medical and Dental University (TMDU), Tokyo, Japan; 2 Department of Human Genetics and Disease Diversity, Graduate School of Medical and Dental Sciences, Tokyo Medical and Dental University (TMDU), Tokyo, Japan; 3 Private University of the Principality of Liechtenstein, Triesen, Liechtenstein; 4 Department of Bioscience and Genetics, National Cerebral and Cardiovascular Center, Suita, Japan; 5 Devision of Arrhythmia, National Cerebral and Cardiovascular Center, Suita, Japan; 6 Omics Research Center, National Cerebral and Cardiovascular Center, Suita, Japan; 7 Department of Cardiovascular Medicine, Hiroshima University, Hiroshima, Japan; 8 Department of Cardiology, International University of Health and Welfare Narita Hospital, Narita, Japan; 9 Department of Cardiovascular Medicine, Kanazawa University Graduate School of Medical Sciences, Kanazawa, Japan; 10 Department of Cardiology, Faculty of Medicine, University of Tsukuba, Tsukuba, Japan; 11 Department of Cardiovascular Medicine, Gunma University Graduate School of Medicine, Maebashi, Japan; 12 Department of Cardiovascular Medicine, Niigata University Graduate School of Medical and Dental Sciences, Niigata, Japan; 13 Department of Human Genetics, Atomic Bomb Disease Institute, Nagasaki University, Nagasaki, Japan; 14 Institute of Education, Tokyo Medical and Dental University (TMDU), Tokyo, Japan; 15 Artificial Intelligence Center for Health and Biomedical Research, National Institutes of Biomedical Innovation, Health and Nutrition, Ibaraki, Japan; 16 Department of Genomic Medicine, National Cerebral and Cardiovascular Center, Suita, Japan; 17 Department of Infection and Host Defense, Graduate School of Medicine, Chiba University, Chiba, Japan; 18 Department of Cardiovascular Medicine, Graduate School of Medicine, Kyoto University, Kyoto, Japan; 19 Center for Arrhythmia Research, Northwestern University Feinberg School of Medicine, Chicago, Illinois, United States of America; 20 Department of Cardiology, National Hospital Organization Okayama Medical Center, Okayama, Japan; 21 Department of Cardiology, Sakakibara heart institute of Okayama, Okayama, Japan; 22 Department of Cardiovascular Therapeutics, Okayama University Graduate School of Medicine, Dentistry and Pharmaceutical Sciences, Okayama, Japan; 23 Division of Advanced Preventive Medical Sciences and Leading Medical Research Core Unit, Nagasaki Univerisity Graduate School of Biomedical Sciences, Nagasaki, Japan; 24 Laboratory for Medical Science Mathematics, Department of Biological Sciences, Graduate School of Science, The University of Tokyo, Tokyo, Japan; 25 Laboratory for Medical Science Mathematics, RIKEN Center for Integrative Medical Sciences, Yokohama, Japan; 26 Department of Cardiovascular Medicine, Shiga University of Medical Science, Otsu, Japan; 27 Department of Cardiovascular Medicine, Nippon Medical School, Tokyo, Japan; Ohio State University, UNITED STATES

## Abstract

Long QT syndrome (LQTS) is one of the most common inherited arrhythmias and multiple genes have been reported as causative. Presently, genetic diagnosis for LQTS patients is becoming widespread and contributing to implementation of therapies. However, causative genetic mutations cannot be detected in about 20% of patients. To elucidate additional genetic mutations in LQTS, we performed deep-sequencing of previously reported 15 causative and 85 candidate genes for this disorder in 556 Japanese LQTS patients. We performed in-silico filtering of the sequencing data and found 48 novel variants in 33 genes of 53 cases. These variants were predicted to be damaging to coding proteins or to alter the binding affinity of several transcription factors. Notably, we found that most of the LQTS-related variants in the RYR2 gene were in the large cytoplasmic domain of the N-terminus side. They might be useful for screening of LQTS patients who had no known genetic factors. In addition, when the mechanisms of these variants in the development of LQTS are revealed, it will be useful for early diagnosis, risk stratification, and selection of treatment.

## Introduction

Long QT syndrome (LQTS) is an arrhythmia disorder characterized by QT interval prolongation in the electrocardiogram (ECG). The QT interval indicates the duration of contraction and relaxation of cardiac ventricles. A prolonged QT interval is caused by the delayed repolarization after contraction of the cardiac ventricles and leads to syncope, ventricular fibrillation, and sudden cardiac death [1]. One in 2500 people has LQTS and familial aggregation has been reported [1, 2].

Previously, 15 causative genes for LQTS have been detected, and recently, *CALM3* and *TRDN* have been newly added [3]. About 75% of the cases carry mutations in either of three ion channel genes, *KCNQ1*, *KCNH2*, or *SCN5A*. Another 5% have known variants in genes encoding beta-subunits of ion channels, multichannel interacting factors, scaffold proteins, or anchoring proteins [4], while causative genetic variants cannot be detected in approximately 20% of LQTS patients [5]. Mutations in any of these causative genes may alter function of corresponding ion channels, which control the action potential of cardiomyocytes. Hyperactivity of sodium and calcium channels or hypoactivity of potassium channels cause prolongation of action potentials, resulting in a long QT interval [6]. The most frequently detected mutations in the LQTS -related genes, *KCNQ1*, *KCNH2*, and *SCN5A* show a strong genotype-phenotype relationship, with a high corresponding risk of symptoms, which is one of the reasons why effective therapeutic options for mutations in these genes have been thoroughly investigated [7]. Other than the three major LQTS causative genes, ten minor genes (*AKAP9*, *CACNA1C*, *CALM1*, *CALM2*, *CAV3*, *KCNE1*, *KCNE2*, *KCNJ2*, *KCNJ5*, *SCN4B*, *SNTA1*) and two atypical type of LQTS causative genes (*ANK2* [8] and *KCNJ2* [9]) have been discovered to date, all of which were tested for within this study [10, 11].

However, similar to other Mendelian diseases, LQTS patients within our cohort exhibited incomplete penetrance [12]. That is, not all carriers with these genetic mutations were equally symptomatic or showing similar phenotypes [13]. These variabilities are considered to be due contributions by environmental factors and/or additional modifying genetic factors with small effect sizes [14].

To explore additional LQTS related genetic factors, several genomic analyses have been performed [15–17]. In a previous study [18], we performed whole-exome sequencing analyses and identified 85 novel candidate genes. The study results suggested that in 37.1% of patients, who did not have mutations in known causative genes, LQTS may be explained by novel genetic factors. Also, protein-protein interaction network analysis (PPI) categorized 10 out of 85 novel candidate genes into a network and half of these were predicted to interact with calmodulin [18]. In addition, further investigation revealed that 87.5% of newly identified mutations in unrelated LQTS patients were in calmodulin-interacting genes [18]. Calmodulin is a multifunctional calcium-binding protein expressed ubiquitously in all eukaryotic cells. Calmodulin senses intracellular calcium levels and plays an important role in calcium signal transduction pathways [19]. In the myocardium, calmodulin interacts with a variety of proteins including those encoded by known causative genes of LQTS and is involved in the control of the cardiac contraction cycle [20]. Additionally, mutations in the calmodulin gene itself have previously been reported to be responsible for ventricular tachycardia and sudden cardiac death [21]. Although the remaining genes identified in our previous study were not classified into specific categories by PPI analysis, their function might have been involved in LQTS development. Therefore, it seems reasonable to think that there may be additional novel risk factors in the 85 candidate genes identified in our previous study. In this study, we performed deep sequencing on 15 known and 85 candidate LQTS genes in 556 LQTS patients to identify additional novel LQTS related variants.

## Materials and methods

### Study subjects

Japanese patients with negative genetic tests for known variants of LQT1, LQT2, and LQT3 [22] were recruited at 21 medical institutes. Based on the Schwartz score [22], we calculated the score for each patient as follows; 3 points: QTc ≥ 480 ms, 2 points: QTc ≥ 460 ms, 1 point: QTc ≥450 ms in males, *Torsade de pointes* observed, syncope observed, familial history of LQTS. We analyzed 556 patients with 2 points or more scores as definite or probable LQTS patients. This study was reviewed and approved by the ethics committees of Nagasaki University, Shiga University of Medical Science, Niigata University, Nippon University, Nippon Medical School, University of Tsukuba, Okayama University, Gunma University, Kyoto University, Kanazawa University, Hiroshima University, Keio University, Saiseikai Kawaguchi General Hospital, Tokyo Saiseikai Central Hospital, Saitama City Hospital, The Jikei University School of Medicine, National Hospital Organization Tokyo Medical Center, Dokkyo Medical University Saitama Medical Center, Matsudo City General Hospital, National Cerebral and Cardiovascular Center, and Tokyo Medical and Dental University (G2000-225). According to the standards of the Declaration of Helsinki, all participants have provided written informed consent prior to any clinical or genetic investigations. Peripheral blood was collected in EDTA test tubes from the patients whose informed consent was obtained and genomic DNA was extracted using a commercially available DNA extraction kit.

### Customized next generation sequencing panel design

Target deep sequencing was performed using peripheral blood DNA preparations. Using the cloud-based software program Ion AmpliSeqTM Designer v2.2.1 (Thermo Fisher Scientific, Waltham, MA, USA), we designed a custom sequencing panel. This panel was designed to amplify 100 genetic loci (15 known and 85 candidate genes; Table 1) that have been established or have been found to be associated with congenital LQTS based on the data obtained in the previous study by Shigemizu et al. [18], including 1000 bp upstream sequence of the first exon and 50 bp intronic sequence before and after each exon. Each of the corresponding primers of the 100 target genes was synthesized by the manufacturer (Thermo Fisher Scientific, Waltham, MA, USA).

**Table 1:** List of 100 target genes explored in this study.

**15 causative genes**				
AKAP9	CALM1	KCNE1	KCNJ2	SCN4B
ANK2	CALM2	KCNE2	KCNJ5	SCN5A
CACNA1C	CAV3	KCNH2	KCNQ1	SNTA1
**85 candidate genes**				
ABCF1	FGF2	NET1	REM1	TNFRSF6B
AKAP8	FSIP2	NLRP13	RIMS1	TOP2A
ANKRD31	GPATCH2	NLRX1	RYR2	TRHDE
ARHGAP22	GUF1	NOV	SDC1	TRMU
ARL13B	HK3	NR5A2	SHANK3	UBR4
ARVCF	HKDC1	NRIP1	SIDT1	UBR5
ATL3	HNRNPM	PI4KA	SIRT6	UBR7
BAIAP3	INTS8	PIK3CG	SLC2A5	UPP1
CCDC168	KIF11	PKD1L2	SLC6A17	USP19
CD276	KIF21B	PLCB4	SNAPC4	VSX1
CELSR1	LRBA	POLRMT	SNAPC5	WDR25
CIT	LRRC8E	PROKR1	SND1	WDR26
CTRL	MDN1	PRSS12	STK32B	WWC2
DIP2A	MESDC1	PRSS57	SYK	YME1L1
DNA2	MKI67	PTOV1	TCTN3	ZNF174
ELMOD2	MYBPHL	PZP	TDRD6	ZNF341
ERAP1	MYLK4	RALGAPA1	TGFBRAP1	ZNF862

### Targeted deep sequencing

Gene libraries of each sample were prepared using Ion AmpliSeq Library Kit for Chef DL8 (Thermo Fisher Scientific, Waltham, MA, USA) according to the manufacturer’s protocol. Library preparation, template generation, and chip loading were automatically completed by using the Ion Chef System (Thermo Fisher Scientific, Waltham, MA, USA). The prepared chip (Ion PI Chip v3, Thermo Fisher Scientific, Waltham, MA, USA) was attached to the sequencer and underwent sequencing using the Ion Torrent Proton Sequencer (Thermo Fisher Scientific, Waltham, MA, USA) and Ion PI HI-Q Chef Kit. The acquired sequencing data was transferred to a server using Ion Torrent Suite (Version 5.0.4; Thermo Fisher Scientific, Waltham, MA, USA).

### Bioinformatics

Raw sequencing reads were analyzed and filtered according to their Phred quality score and the minimum coverage. Minimum Phred quality score and coverage were set for 15 and 5, respectively [23]. Variants were detected by the Ion Torrent Variant Caller program. ANNOVAR was used to perform the functional annotation of variants, using GRCh37 assembly as a reference [24]. To find patients’ specific variants in an exon, the following filtering was performed: 1) variants located in exonic regions were extracted, 2) synonymous variants were excluded, 3) variants which are not described in several public databases (ExAC: Exome Aggregation Consortium, ESP6500: NHLBI Go Exome Sequencing Project, HGVD: Human Genetics Variation Database, jMorp: Japanese Multi Omics Reference Panel 14KJPN) or the minor allele frequency was less than 0.001 were extracted, then 4) functional effects of the altered protein structure of variants were predicted using the scores of PolyPhen-2 (http://genetics.bwh.harvard.edu/pph2/) [25], Sorting Intolerant From Tolerant (SIFT; http://sift.jcvi.org/www/) [26], Combined Annotation Dependent Depletion (CADD; https://cadd.gs.washington.edu/) [27], Protein Variation Effect Analyzer (PROVEAN; http://provean.jcvi.org/index.php) [28, 29] and CONsensus DELeteriousness score (CONDEL; http://bbglab.irbbarcelona.org/fannsdb/home) [30]. According to the definition of each algorithm, cut off scores were set to <0.05 for SIFT, >0.5 for polyphen-2, >20 for CADD, <-2.5 for PROVEAN and >0.49 and label “D” for CONDEL. When the amino acid positions indicated by the algorithms did not match the public databases, we excluded these variants from further consideration. Variant information was obtained from the annotation file output by ANNOVAR, Ensembl (https://ensembl.org/index.html), and NCBI (https://www.ncbi.nlm.nih.gov/).

To find LQTS specific variants in the upstream region, the following filtering was performed: 1) variants located in upstream regions were extracted, 2) single nucleotide variants were extracted, and 3) variants predicted to have affinity change for transcription factors (TFs) by transcription factor affinity prediction (TRAP; http://trap.molgen.mpg.de/cgi-bin/home.cgi) [31]. Forty-one sequences, including target SNP and ± 20 bases were applied to sTRAP with the following settings: matrix file; transfac_2010.1 vertebrates, background model; human_upstreams, multiple test correction; Benjamini-Hochberg. A list of TFs with a reference or alternative P-value < 0.01 and in the top ten rankings were queried gene ontology terms of these TFs by Comparative Toxicogenomics Database (CTD; http://ctdbase.org/tools/batchQuery.go) [32]. Known variants on the 15 LQTS causative genes (Table 1) were obtained from dbSNP (https://www.ncbi.nlm.nih.gov/snp/) and known LQTS related variants were listed. These variants were compared to the variants of all sample files. If the patients had these variants, the pathogenesis information of these variants was checked in the dbSNP and ClinVar (https://www.ncbi.nlm.nih.gov/clinvar/) and excluded from this study [33–48]

### Experimental validation

To validate potentially disease-causing variants detected *in silico*, we performed Sanger sequencing. Primer sets were individually tested for the optimal annealing temperature of each primer using two separate control DNA samples and a DNA-free negative control. Ten nanograms of genomic DNA were amplified using Taq DNA polymerase according to the manufacturer’s manuscript (Genscript, Piscataway, NJ, USA). Two microliters of dimethyl sulfoxide (DMSO) were added if necessary (Sigma-Aldrich, St. Louis, MI, USA). Thermal cycling (Veriti, Applied Biosystems, Foster City, CA, USA) was performed at 94°C for 2 minutes, followed by 35 cycles of 94°C for 30 seconds, appropriate annealing temperature for 30 seconds, and 72°C for 40 seconds. Two microliters of PCR products were electrophoresed on 0.8% agarose gel contains 0.5 μg/ml of ethidium bromide along with 5 μl of 1 Kb of DNA ladder (Nippon Genetics Europe GmBH, Düren, NRW, Germany). PCR product bands were quantified by LAS4000 luminescent image analyzer (General Electric, Boston, MA, USA). To inactivate residual dNTPs and primers, 2.5 μl of PCR product and 1 μl of ExoSAP-IT reagent (Affymetrix, Santa Clara, CA, USA) were mixed and incubated at 37°C 15 minutes, followed by 80°C for 15 minutes. Each PCR product was diluted with 136.5 μl of PCR grade water (Millipore, Billerica, MA, USA) and 2 μl was provided for Sanger sequencing. Sanger sequencing was performed using BigDye Terminator v3.1 Cycle Sequencing kit (Applied Biosystems, Foster City, CA, USA) according to the manufacturer’s protocol. The primer list is shown in S1 and S2 Tables.

## Results

We performed deep sequencing focusing on 15 causative [49] and 85 candidate genes [18] of LQTS in 556 Japanese LQTS patients (Tables 1 and 2). Extracted variants were validated by Sanger sequencing, and we found 48 novel variants that are possibly associated with LQTS in either the exon or upstream regions (Table 3 and 4).

**Table 2:** The average data output in deep sequencing.

Number of samples	Detected bases	Mean read length (bp)	Mapped reads	On target region	Mean depth	Detected variants
556	29,83,23,362	119	24,85,134	98.21%	473.5	565

**Table 3:** Characteristics of newly detected variants and their functional prediction scores.

Gene	Chr. No	Position	Transcript ID	Coding sequence change	Protein change	Function prediction algorithm					Number of detected Patients
						SIFT	PolyPhen-2	CADD PHRED	PROVEAN	CONDEL	
**causative genes**											
**KCNQ1**	11	2604719	NM_000218	c.976A>G	p.K326E	0.01	0.995	27.8	-3.4	0.63	1
		2797199	NM_000218	c.1600A>G	p.K534E	0	1	27.8	-3.42	0.65	2
**CACNA1C**	12	2717835	NM_001129827	c.3575A>T	p.Q1192L	0	0.981	26.8	-6.2	0.68	1
		2783752	NM_001129827^a^	c.4916A>C	p.K1639T	0	0.76	27.2	-5.54	0.59	2
**SCN5A**	3	38648189	NM_000335^a^	c.1111C>G	p.Q371E	0	0.895	23.6	-2.94	0.71	2^b^
**ANK2**	4	114238868	NM_001148^a^	c.2699G>C	p.R900P	0.01	0.914	31.0	-3.92	0.55	1
**KCNH2**	7	150671904	NM_000238^a^	c.202T>A	p.F68I	0	1	27.5	-3.56	0.64	1
**KCNJ5**	11	128781760	NM_000890	c.592G>A	p.E198K	0.01	0.998	25.6	-3.17	0.55	1
**candidate genes**											
**RYR2**	1	237659869	NM_001035	c.2020T>C	p.Y674H	0	1	27.9	-3.99	0.77	1
		237890481	NM_001035	c.10820C>G	p.P3607R	0	1	26.6	-7.65	0.63	1
		237936938	NM_001035	c.11765A>G	p.E3922G	0	1	32.0	-6.14	0.69	1
		237947013	NM_001035	c.12001G>T	p.D4001Y	0	1	29.5	-7.94	0.60	1
**LRBA**	4	151356802	NM_006726^a^	c.7013C>T	p.R2338Q	0	0.998	32.0	-3.04	0.79	1
		151412117	NM_006726^a^	c.6434C>T	p.R2145H	0	1	32.0	-4.1	0.64	1
		151835430	NM_006726^a^	c.1078G>T	p.Q360K	0.01	0.943	32.0	-3.52	0.58	1
**CELSR1**	22	46786320	NM_014246	c.6314G>A	p.T2105I	0	1	26.1	-5.78	0.54	1
		46931273	NM_014246	c.1795G>A	p.R599W	0	0.991	23.6	-6.17	0.53	1
**ARHGAP22**	10	49663152	NM_021226	c.685A>T	p.S229T	0.01	0.999	24.7	-2.73	0.57	1
**CTRL**	16	67964104	NM_001907	c.617G>A	p.G206D	0	1	26.3	-6.41	0.66	1
**ERAP1**	5	96136666	NM_016442	c.562T>C	p.R188G	0	0.995	25.9	-6.34	0.61	1
**LRRC8E**	19	7965764	NM_001268284^a^	c.2357C>T	p.P786L	0	1	27.3	-6.57	0.60	1
**PIK3CG**	7	106509069	NM_001282426^a^	c.1063T>C	p.W355R	0	0.604	24.9	-11.91	0.58	1
**RALGAPA1**	14	36226078	NM_194301	c.584G>T	p.P195H	0	1	26.5	-7.35	0.64	1
**RIMS1**	6	73023351	NM_014989	c.4106C>G	p.S1369C	0.04	0.999	28.8	-2.83	0.65	1
**SDC1**	2	20405113	NM_001006946	c.139A>C	p.S47A	0	0.992	25.6	-2.63	0.54	1
**SIDT1**	3	113344964	NM_017699	c.2323T>G	p.S775A	0	1	26.8	-2.7	0.58	1
**TNFRSF6B**	20	62328383	NM_003823	c.263G>A	p.C88Y	0	1	26.7	-10.18	0.71	1
**WDR25**	14	100992303	NM_001161476^a^	c.1198C>T	p.R400W	0	1	26.2	-5.51	0.58	1
**ZNF174**	16	3452278	NM_001032292^a^	c.274G>A	p.E92K	0	0.997	29.7	-3.48	0.56	1

The amino acid positions were from the results of each algorithm or Ensembl. DNA positions and transcript ID were from ANNOVAR or Ensembl.

^a^These variants were on the multiple forms of transcripts. The top mRNA ID of NCBI RefSeq is indicated.

^b^This variant was detected in siblings.

**Table 4:** Variants in the upstream region that could alter the binding affinity of transcription factors predicted by TRAP.

Gene	Chr. No	Position	Ref	Alt	TF^a^	GoTerm ID^b^	GoTermName^b^	P-value		Difference^c^	Number of detected Patients
								P_Alt_	P_Ref_		
**causative genes**											
**SNTA1**	20	32031723	G	A	SRF	GO:0007507	heart development	0.052	0.001	-1.896	1
						GO:0055003	cardiac myofibril assembly				
		32032568	C	A	GATA6	GO:0003007	heart morphogenesis	0.857	0.001	-3.016	1
						GO:0010666	positive regulation of cardiac muscle cell apoptotic process				
						GO:0060045	positive regulation of cardiac muscle cell proliferation				
					GATA3	GO:0061026	cardiac muscle tissue regeneration	0.396	0.007	-1.762	
						GO:0003215	cardiac right ventricle morphogenesis				
**KCNQ1**	11	2465486	G	C	HES1	GO:0003143	embryonic heart tube morphogenesis	0.227	0.005	-1.656	2
						GO:0010667	negative regulation of cardiac muscle cell apoptotic process				
**ANK2**	4	113739117	A	G	POU5F1	GO:0003130	BMP signaling pathway involved in heart induction	0.480	0.001	-2.553	1
						GO:0060913	cardiac cell fate determination				
**CAV3**	3	8775224	G	A	ZIC1	GO:0021592	fourth ventricle development	0.009	0.277	1.470	1
**candidate genes**											
**NET1**	10	5453591	T	G	SMAD1	GO:0060038	cardiac muscle cell proliferation	0.075	0.000	-3.651	5
		5454100	T	C	POU6F1	GO:0007507	heart development	0.016	0.000	-2.095	1
**PRSS12**	4	119274873	C	G	PPARA	GO:0007507	heart development	0.050	0.005	-1.041	3
**CD276**	15	73976230	G	T	PPARA	GO:0007507	heart development	0.010	0.076	0.896	1
**CTRL**	16	67966622	G	A	NF1	GO:0007507	heart development	0.000	0.000	1.604	1
**HNRNPM**	19	8508971	T	C	GATA3	GO:0061026	cardiac muscle tissue regeneration	0.348	0.002	-2.350	1
						GO:0003215	cardiac right ventricle morphogenesis				
**KIF21B**	1	200993473	A	G	VDR	GO:0007507	heart development	0.000	0.029	3.529	1
**MYLK4**	6	2751237	G	A	TAL1	GO:0007507	heart development	0.008	0.053	0.834	1
**NLRP13**	19	56444514	A	G	GATA3	GO:0061026	cardiac muscle tissue regeneration	0.003	0.050	1.292	1
						GO:0003215	cardiac right ventricle morphogenesis				
					SMAD4	GO:0003161	cardiac conduction system development	0.027	0.004	-0.832	
						GO:0010614	negative regulation of cardiac muscle hypertrophy				
						GO:0010666	positive regulation of cardiac muscle cell apoptotic process				
**SIRT6**	19	4182605	G	A	SP4	GO:0008016	regulation of heart contraction	0.146	0.008	-1.248	1
**PKD1L2**	16	81254548	T	C	ZIC3	GO:0001947	heart looping	0.005	0.076	1.222	1
**UBR4**	1	19537075	A	C	VDR	GO:0007507	heart development	0.010	0.000	-3.752	1
		19537026	C	T	PPARG	GO:0007507	heart development	0.009	0.101	1.040	1

^a^When the same TF was listed, only the top information was indicated.

^b^Difference was calculated by the following formula: log(P_Ref_)-log(P_Alt_).

^b^Representative categories are indicated.

^c^Difference was culculated by the following fomula: log(P_Ref_)-log(P_Alt_).

We found nine novel variants on exon regions of six known causative genes. In addition to that, we also found 21 novel variants on exon regions of 15 candidate genes (Table 3). They were inferred to have functional damage to each of the respectively coded proteins by functional prediction algorithms. Interestingly, among the known causative genes, we found three novel variants in *KCNQ1*, two novel variants in *CACNA1C*, one novel variant in *SCN5A*, *ANK2*, *KCNH2* and *KCNJ5* (Table 3). Among candidate genes, multiple mutations were detected in *RYR2*, *LRBA*, and *CELSR1* (Table 3).

In the upstream region, we searched the variants which predicted to change the binding affinity for TFs in these genes and are involved in cardiac function using TRAP algorithm and CTD, respectively. As a result, we found four and 18 variants in causative and candidate genes, respectively (Table 4).

## Discussion

In this study, we performed target deep sequencing of Japanese LQTS patients and found novel variants predicted to alter the biological function of the affected genes. We found three novel possibly pathogenic variants in the coding region of *KCNQ1* (Table 3). One of these variants, c.785T>C changes the amino acid located at the boundary point of the cytosolic site of the fifth transmembrane (p.L262P), while a variant that changes the leucine to valine (p.L262V) has been reported as an LQTS-related variant previously [43, 50]. Since the structure of proline is quite different from that of leucine or valine, the function of p.L262P is expected to be more damaging compared to p.L262V, indicating that this variant is likely a strong causative candidate for LQTS.

We found one novel variant in the exon region of *SCN5A* in two siblings (c.1111C>G, p.Q371E, Table 3). *SCN5A* codes for a voltage-gated sodium channel subunit and regulates the initial upstroke of the action potential [51]. The variant is located at the pore-forming domain between the fifth and sixth transmembrane, suggesting to have a great influence on the function of SCN5A as an ion channel.

We found one novel variant in *KCNH2* (c.202T>A, p.F68I), which encodes one of the alpha-subunit of the potassium channel and regulates the final repolarization of the cardiac action potential [52]. At this position, a variant which changes phenylalanine to leucine (c.202T>C, p.F68L) was reported in an LQTS patient [53]. This variant is located at the Per-Arnt-Sim (PAS) domain of the N-terminus cytosolic region of KCNH2, and mutation on the PAS domain has been suggested to alter the channel activity [54].

Among the minor causative LQTS genes, we found two novel variants in the exons of *CACNA1C*. *CACNA1C* encodes a calcium ion channel playing an important role in excitation and contraction of the heart, contributing to normal heart development and regulation of heart rhythm. We found two variants in *CACNA1C*, one of which was located in the intracellular loop linking repeat III and IV (c.3575A>T; p.Q1192L). Several variants located in the cytosolic loop have been reported as LQTS-related ones, suggesting that gain-of-function mutations affect the function of *CACNA1C* [5, 55, 56].

We found a novel variant in *ANK2*, located between the 24th ANK repeat and ZU5 domain (c.2699G>C, p.R900P). As the ANK repeat has a critical role in the normal electric activity of the heart, the novel variant might alter the maintenance of ion channels and transporters and affect cardiac function [57, 58].

*KCNJ5* encodes a subunit of the potassium channel controlled by G-proteins and allows potassium ions into cells. We found one novel variant (c.592G>A, p.E198K) in the C-terminus cytosolic domain of *KCNJ5*. Previously, only one variant (p.G387R) was detected in *KCNJ5* in a Chinese family affected by LQTS [48]. This variant was also located in the C-terminus cytosolic domain and indicated a loss of function phenotype. The novel variant found in our present study might also contribute to the development of LQTS by a similar mechanism.

**Fig 1:**
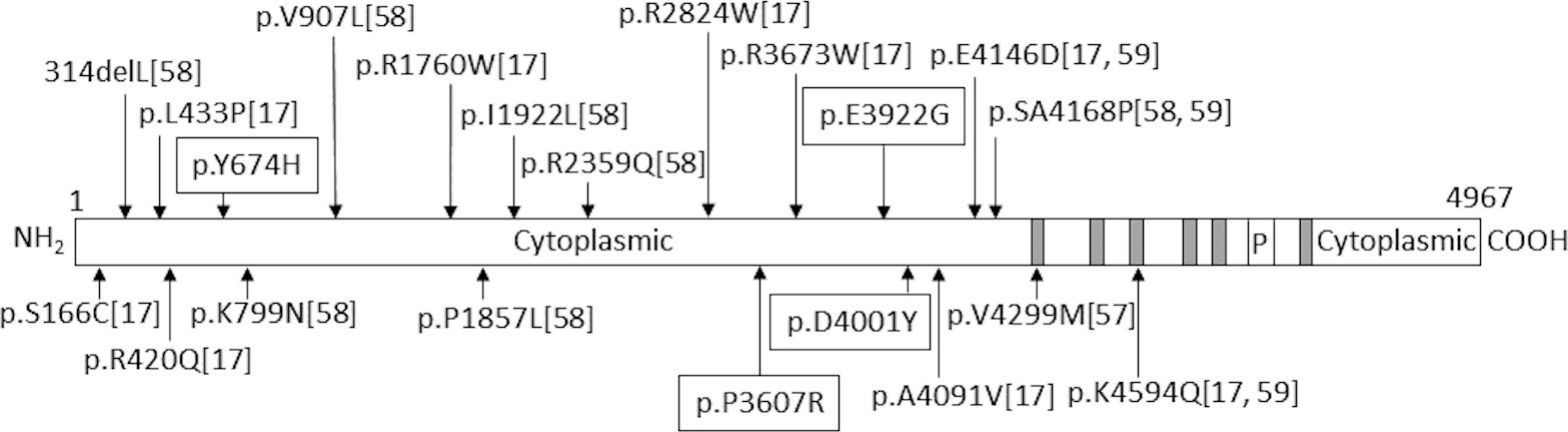
Schematic view of RYR2 mutations in long QT syndrome. Only those predicted to be damaging *in silico* are shown. The number in the parentheses indicates the reference number in the manuscript. Those with surrounding lines are the novel variants identified in this study. Gray boxes indicate the transmembrane region and “P” indicates the pore-forming region.

In this study, four different LQTS-related variants in *RYR2* were identified. *RYR2* is a component of the calcium channel and supplies calcium to the myocardium in response to ryanodine concentration [59]. Although several LQTS-related variants on *RYR2* have been reported [18, 60, 61], a considerable part of *RYR2* variants are shared with other cardiac diseases, and it is particularly considered as a causative gene for catecholaminergic polymorphic ventricular tachycardia (CPVT). Miyata et al. divided the variants found in *RYR2* of CPVT and LQTS patients into N-terminal (1–2177 a.a.), central (2178–4075 a.a.), and C-terminal (4076–4959 a.a.) regions and found no significant positional differences between these diseases [61]. They suggested that patients with *RYR2* variants and bradycardia should be carefully diagnosed to avoid misdiagnosing CPVT patients as LQTS patients [61]. Hirose et al. revealed *RYR2* loss-of-function mutations causing QT prolongation [62]. We found that most LQTS-related variants reported in previous studies [18, 42, 48, 63] and in our present study were located on the large cytosolic region of the N-terminal side (Fig 1). Therefore, we suppose that clarifying the location and the type of dysfunction in *RYR2* in detail might help to distinguish between LQTS and CPVT. Further analysis may reveal which *RYR2* variants are important for the development of LQTS.

Among the other candidate genes, *LRBA* showed three novel variants in this study (Table 3). LRBA is known to be involved in trafficking endosomes containing ligand-activated receptors [64, 65]. Many LQTS-related ion channel proteins are transported and recruited to the plasma membrane by endosomal trafficking [66, 67]. Two novel variants in *LRBA* (c.7013C>T, p.R2338Q and c.6434C>T, p.R2145H) were located in the BEACH domain which plays an important role in endosomal vesicle trafficking [68]. Many LQTS-related ion channel proteins are transported and recruited to the plasma membrane by endosomal trafficking and the formation of functional complexes [66, 67]. Therefore, LRBA could also possibly contribute to the maintenance of LQTS-related membrane proteins, and variants that alter the function of LRBA might contribute to the pathogenesis of LQTS.

In the upstream region of the gene, we found variants predicted to alter the binding affinity for TFs (Table 4). These TFs were annotated to be involved in the pathway of heart development, morphogenesis, and cell proliferation in GO term name [32]. If these TFs truly regulate the downstream genes examined in this study, it will alter the expression level of each gene product which is related to the function of ion channels and/or calmodulin. Inadequate expression of these proteins might affect the proper function of cardiac cells and lead to development of LQTS [69].

It is worth noting that one variant in the NET1 gene (chr10: 5453591) was shared with five patients (Table 4). NET1 is known to control Rho signaling [70, 71]. Rho signaling play an important role in the cardiac system [72] suggesting the variant we found in this study might influence the development of LQTS.

Recently, Adler et al. advocated that the known causative variants for LQTS have not been well experimentally validated and reported that among 17 reported causative genes for LQTS, only three (*KCNQ1*, *KCNH2*, *SCN5A*) were definitive genes for typical LQTS [3]. In our study, we found novel variants in six out of 15 causative genes and half of them were in the definite LQTS causative genes. However, among three definite LQTS genes, *KCNQ1* and *KCNH2* are also reported as causative genes for the short-QT syndrome [73]. Depending on the locus of mutations in the cardiac channel genes, different phenotypes can be observed. For example, the loss-of-function mutation in *KCNQ1* causes prolongation of QT interval [74], and the gain-of-function mutation in *KCNQ1* causes shortening of QT interval [75]. Because all novel variants found in this study are predictive, functional analyses and their validation remain to be performed to prove that these are truly causative variants/genes for LQTS. Revealing the effect of each variant on the onset of the disease would be expected to screen the potential LQTS patients at an early stage. In addition, genetic diagnoses of LQTS can be used for screening patients’ family members and contributes to the implementation of therapies for high-risk relatives [11]. We believe the elucidation of novel variants related to LQTS will improve diagnosis, risk stratification, and early treatment of LQTS patients in the future, especially for those who had previously failed the genetic screening.

## Supporting information

S1 Table List of primer sequences used for SNP validation on exon region. (XLSX)

S2 Table List of primer sequences used for SNP validation on upstream region. (XLSX)
