# Correction: Targeted deep sequencing analyses of long QT syndrome in a Japanese population

**DOI:** 10.1371/journal.pone.0315106

**Published:** 2024-12-03

**Authors:** Yuki Nagata, Ryo Watanabe, Christian Eichhorn, Seiko Ohno, Takeshi Aiba, Taisuke Ishikawa, Yukiko Nakano, Yoshiyasu Aizawa, Kenshi Hayashi, Nobuyuki Murakoshi, Tadashi Nakajima, Nobue Yagihara, Hiroyuki Mishima, Takeaki Sudo, Chihiro Higuchi, Atsushi Takahashi, Akihiro Sekine, Takeru Makiyama, Yoshihiro Tanaka, Atsuyuki Watanabe, Motomi Tachibana, Hiroshi Morita, Koh-ichiro Yoshiura, Tatsuhiko Tsunoda, Hiroshi Watanabe, Masahiko Kurabayashi, Akihiko Nogami, Yasuki Kihara, Minoru Horie, Wataru Shimizu, Naomasa Makita, Toshihiro Tanaka

In Fig 1, the citation numbers are incorrect. Please see the correct Fig 1 here.

**Fig 1:**
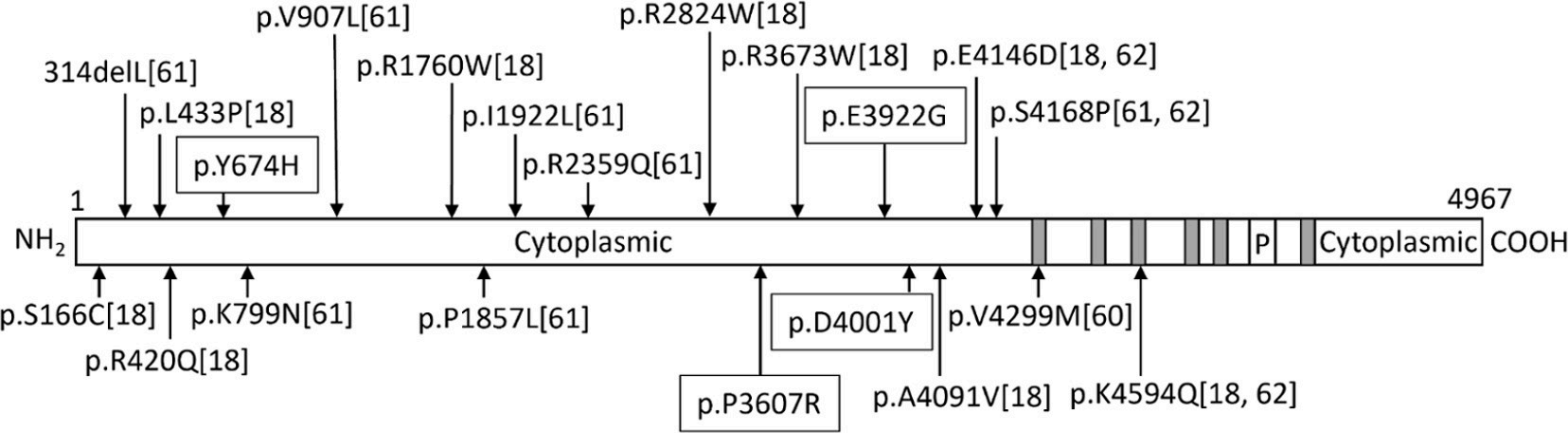
Schematic view of RYR2 mutations in long QT syndrome. Only those predicted to be damaging in silico are shown. The number in the parentheses indicates the reference number in the manuscript. Those with surrounding lines are the novel variants identified in this study. Gray boxes indicate the transmembrane region and “P” indicates the pore-forming region.

## References

[1] Nagata Y, Watanabe R, Eichhorn C, Ohno S, Aiba T, Ishikawa T, et al. (2022) Targeted deep sequencing analyses of long QT syndrome in a Japanese population. PLoS ONE 17(12): e0277242. doi: 10.1371/journal.pone.0277242 36480497 PMC9731492

